# MangoLeafNet-XAI: an attention-enhanced deep learning architecture for accurate and interpretable mango leaf disease classification

**DOI:** 10.3389/fpls.2026.1776537

**Published:** 2026-03-09

**Authors:** Md. Abdur Rahman, Md. Tofael Ahmed Bhuiyan, Farzan Majeed Noori, Md Zia Uddin, Abdul Kadar Muhammad Masum

**Affiliations:** 1 Computational Intelligence Lab, Southeast University, Dhaka, Bangladesh; 2 Department of Informatics, University of Oslo, Oslo, Norway; 3 Sustainable Communication Technologies Department, SINTEF Digital, Oslo, Norway; 4 Department of Computer Science and Engineering, Southeast University, Dhaka, Bangladesh

**Keywords:** agricultural automation, attention mechanism, deep learning, Densenet, ensemble learning, explainable AI, Grad-CAM, lime

## Abstract

A critical challenge in agricultural automation is the precise detection of mango leaf diseases that compromise crop quality and yield. To address the limitation of existing heavy models in resource-constrained agricultural environments, this study proposes MangoLeafNet-XAI, a novel lightweight deep learning architecture. The model synergistically integrates Efficient Channel Attention (ECA) modules with a DenseNet-121 backbone to adaptively refine features and capture subtle pathological patterns with high precision. The proposed framework was rigorously evaluated using a 5-fold cross-validation and soft-voting ensemble strategy across three public datasets (MLDID, Mango Leaf Disease, and Harumanis). These datasets encompass diverse environmental conditions and distinct disease classes, including Anthracnose, Bacterial Canker, Die Back, Gall Midge, Powdery Mildew, Sooty Mould, and Cutting Weevil. MangoLeafNet-XAI achieved state-of-the-art accuracies of 98.83% on MLDID, 98.09% on the Mango Leaf Disease Dataset, and 98.76% on the Harumanis dataset. A primary contribution of this work is the optimal balance between performance and computational efficiency, utilizing only 6.9 million parameters, making it highly suitable for deployment on edge devices. Moreover, the interpretability of AI methods, such as Grad-CAM and LIME, that are used to explain the rationale behind predictions to offer pathological explanations, also validate the focus on clinically important aspects of the model. The results discuss the key limitations of existing methods, such as computational complexity, inability to interpret the findings, and dataset-dependent overfitting, and demonstrate a high level of resilience and generalizability on diverse datasets. MangoLeafNet-XAI will be a new benchmark of reliable, deployable, as well as accurate disease diagnosis systems, in smart agriculture.

## 1 Introduction

The fruit of the *Mangifera indica* L. holds immense value in agriculture and international trade, renowned for its exceptional taste and nutritional benefits (Tharanathan et al., 2006). Foliar diseases, however, represent a critical threat to mango cultivation, imposing tremendous losses in production and decreased fruit quality, despite the crop’s profound economic and cultural significance. Foliar diseases, caused by bacterial, fungal, and viral infections, primarily target the leaves, disrupting photosynthesis mechanisms and compromising the overall plant health. Thus, early and accurate disease detection is vital for maintaining orchard health and sustaining uniform fruit quality (Okigbo and Osuinde, 2003; Hassoon, 2022). Such complexity in these diseases necessitates sophisticated diagnostic and management approaches to circumvent their detrimental effects. Ultimately, the success of such interventions underpins the long-term sustainability and economic viability of the global mango industry.

Conventional diagnosis relies primarily on manual visual inspections by horticultural experts. While conventional, the approach is subjective and prone to variability among observers. Such variations are likely to exacerbate loss of yield, reduce fruit quality, and facilitate spread of disease through delayed intervention. In the recent past, however, machine learning (ML) and computer vision technology have revolutionized plant disease management to develop computerized systems that offer quick, precise, and impartial leaf disease diagnosis (Faye et al., 2022). Such sophisticated frameworks make disease avoidance easy, maximize orchard productivity, and allow for early intervention (Manoharan et al., 2021; Trongtorkid and Pramokchon, 2018).

Among ML techniques, Convolutional Neural Networks (CNNs) outshine image-based disease classification in the case of mango leaf diseases. Hierarchical feature extraction by CNNs neatly distinguishes between healthy and infected leaf images, and it detects subtle and intricate visual characteristics. Their capacity for detecting subtle patterns makes them ideally suited for the intricate task of classifying mango leaf diseases (Arivazhagan and Ligi, 2018). By automating diagnostic procedures, CNN-based systems accelerate disease control, enhance diagnosis accuracy, and encourage more sustainable, cost-effective, and resilient mango cultivation environments.

Albeit improvements in the application of CNN to classify plant diseases, issues of scalability, efficiency, and generalizability are chronic barriers (Salehi et al., 2023). While pre-trained CNNs are powerful for generic image classification, they often underperform in specialized domains like mango leaf disease diagnosis (Das et al., 2025). In these areas, diagnostic accuracy is frequently compromised by an inability to distinguish subtle morphological variations and disease-characteristic features. Besides, training deep networks entails high computation with high-level processing power, memory, and time requirements (Voulodimos et al., 2018; Rawat and Wang, 2017). Such computation-hungry character poses a major bottleneck to field-level practical application, particularly under constrained computation resources common in resource-limited field environments. Consequently, a significant gap remains between theoretical model performance and real-world usability, highlighting the urgent need for domain-specific, low-computation, and lightweight solutions tailored to crop disease dynamics. The innovation of this work lies in addressing the ‘accuracy-interpretability-efficiency’ trilemma. Existing high-accuracy models often rely on massive architectures like VGG-16, which are computationally prohibitive for field-level mobile applications. Furthermore, the absence of explanatory evidence in prior works limits their adoption by agricultural experts who require pathological justification. MangoLeafNet-XAI fills these gaps by introducing a hybrid architecture that maintains a low parameter footprint (6.9M) while utilizing Grad-CAM and LIME to verify that the model is learning genuine disease traits rather than dataset-specific noise. By validating across three geographically distinct datasets, this study offers a recommendation for transitioning from opaque, high-capacity models to transparent, deployable diagnostic systems.

To surmount these constraints, this study introduces MangoLeafNet-XAI, a novel explainable deep learning framework tailored for precise mango leaf disease classification. The proposed design synergistically fuses the adaptive channel-wise feature refinement of Efficient Channel Attention (ECA) modules with the aggressive feature reuse of a DenseNet backbone. The synergy achieves state-of-the-art classification accuracy, finely tuned to subtle disease manifestations. Prominent in our design are priorities of computational frugality, transparency, and efficacy. We employ rigorous 5-fold cross-validation augmented with ensemble techniques for model robustness and generalizability. Furthermore, to bring about credibility in agricultural uses, we incorporate Explainable AI (XAI) mechanisms, offering transparent explanation of decision rationales and making sure that predictions are aligned with verifiable pathological indicators. The unresolved challenge addressed here is the lack of a ‘clinically-transparent’ model that can maintain high diagnostic precision without the computational overhead of traditional deep networks. Most existing mango disease models fail when moved from controlled datasets to the field because they capture spurious background correlations rather than actual disease markers. MangoLeafNet-XAI uniquely bridges this gap by utilizing Efficient Channel Attention to force the model to ‘attend’ to relevant morphological traits, combined with a 5-fold ensemble strategy to ensure cross-dataset robustness. By doing so, it provides a unique architectural blueprint that solves the deployment constraint of high resource demand while simultaneously resolving the trust deficit through evidence-based AI.

In summary, the principal contributions of this work encompass:

- A novel deep learning architecture, MangoLeafNet-XAI, merging DenseNet and Efficient Channel Attention (ECA) for superior mango leaf disease categorization.
- Implementation of stringent 5-fold cross-validation and ensemble averaging, attaining a remarkable 98.83% accuracy on the Mango Leaf Disease Identification Dataset (MLDID), establishing a new benchmark.
- Deployment of Local Interpretable Model-agnostic Explanations (LIME) to prioritize interpretability, surmounting black-box limitations and affirming model recommendations through visual corroboration of genuine disease traits.
- Provision of a deployable asset for smart farming systems, enabling prompt, evidence-driven actions while striking an optimal balance between peak accuracy and modest parameter count (6.9M).

The remainder of the paper is structured as follows. Section 2 provides a comprehensive literature review of deep learning for plant disease classification with particular emphasis on mango leaf detection to highlight the key progress and open issues. Section 3 describes the methodology of MangoLeafNet-XAI, covering data collection procedures, preprocessing steps, architectural designs, loss function engineering, and ensemble methods. Section 4 presents an extensive experimental analysis on three publicly available datasets, with quantitative metrics alongside XAI visualizations and comparisons to state-of-the-art pre-trained models. Section 5 is a critical discussion of the outcomes, including real-world implications for agricultural deployment, model limitations, and factors for effectiveness. Section 6 concludes by summarizing the main findings and giving future research directions.

## 2 Related Works

Convolutional neural networks and deep learning have facilitated great leaps in plant disease classification. Sladojevic et al. (2016) developed a CNN-based model specifically dedicated to plant disease detection that was extremely accurate and rendered CNNs appropriate for discovering subtle disease-specific features from leaf images. Building upon this foundation, Mohanty et al. (2016) demonstrated the revolutionary capability of deep learning in agriculture, revealing improved performance of deep neural networks when compared with conventional approaches due to the capacity of deep networks to learn intricate disease features.

Looking at mango leaf disease, Prabu and Chelliah (2022) suggested a CNN model augmented with crossover-based Levy flight distribution algorithm that achieved 98.42 recall for Mango Anthracnose but with high computational cost. Rizvee et al. (2023) presented LeafNet, a CNN model that was more accurate than VGG16 and AlexNet with 98.55 accuracy and with fewer computational costs such that it could be utilized in low-resource devices. Ferentinos (2018) demonstrated the flexibility of architectures like ResNet and VGGNet in plant pathology identification, while Varma et al. (2025) have identified InceptionV3 to be 99.87 accurate in diagnosing mango leaf disease. Rao et al. (2021) demonstrated a framework based on AlexNet with 89 percent accuracy, drawing attention to mobile diagnostic applications despite limited dataset variability.

To increase efficiency, light-weight CNNs were proposed by Too et al. (2019) and Hari and Singh (2023), the latter achieving 99.14 accuracy with only 101,000 parameters. Robustness was also improved using ensemble learning, as shown by Hang et al. (2024) using Choquet fuzzy integrals to achieve 99.80 accuracy. Distributed computing techniques such as MapReduce improved scalability for large agriculture data (Matrouk et al., 2025). Self-supervised learning (SSL) also emerged to address limited labeled data (Yang et al., 2024, 2023). Recent models achieved high accuracy but lacked interpretability (Mahbub et al., 2023; Singh et al., 2019; Vijay and Pushpalatha, 2023).

The proposed MangoLeafNet-XAI achieves 98.83 accuracy by integrating Efficient Channel Attention with DenseNet and Local Interpretable Model-agnostic Explanations, offering accuracy, efficiency, and explainability for real-world agricultural diagnostics. **Table 1** presents a comparative overview of the deep learning algorithms currently used in plant disease diagnosis, emphasizing their benefits, drawbacks, and contributions.

**Table 1:** Comparative analysis of plant disease classification methods.

Ref.	Model	Dataset	Contribution	Challenges
Sladojevic et al. (2016)	Deep CNN (CaffeNet)	Leaf image dataset	Applied a DL method for accurate plant disease detection from leaf images.	Limited coverage of rare plant diseases, reliance on visible symptoms excluding early stages, lack of real-time field-deployable solutions, and risks of overfitting due to small samples in rare classes.
Mohanty et al. (2016)	AlexNet, GoogLeNet (Transfer Learning)	PlantVillage dataset	Applied DL for accurate plant disease detection from leaf images, enabling smartphone-based diagnosis.	Reliance on visible symptoms, lack of real-time solutions, and overfitting due to small sample sizes. Multi-modal data integration remains a key research gap.
Prabu and Chelliah (2022)	CNN optimized by CROLFD + MobileNetV2 + SVM	Mango leaf dataset (India)	Introduced a CNN-based framework for mango leaf disease classification optimized with a novel algorithm.	Limited validation, unexplored security risks, lack of interpretability and gaps in scalability and domain adaptation.
Rizvee et al. (2023)	LeafNet (CNN)	MangoleafBD dataset	Introduced LeafNet, a lightweight CNN excelling in detecting seven mango leaf diseases.	Limited research and datasets in Bangladesh hinder effective mango leaf disease detection models.
Ferentinos (2018)	VGG (highest accuracy), AlexNetOWTBn	Open database of 87,848 images.	Developed CNN-based DL models for plant disease classification.	Performance drops with new data sources, also limited testing in real conditions.
Varma et al. (2025)	Transfer learning models (InceptionV3 best)	PlantVillage	Identifies InceptionV3 as the best model for mango leaf disease detection.	Need for diverse, real-field, and geographically varied datasets to improve model accuracy in agriculture.
Too et al. (2019)	DenseNet 121, ResNet 50/101/152	PlantVillage (54,306 images)	Analyzed fine-tuning state-of-the-art DL architectures for accurate plant disease classification.	High computational time for ResNet 152 and Inception V4.
Hari and Singh (2023)	CNN	Mixed (Kaggle, Mendeley) for banana, guava, mango	Introduced a lightweight CNN model for banana, guava, and mango leaf disease detection.	Limited to banana, guava, and mango; further validation needed for other leaves.
Hang et al. (2024)	Fuzzy Ensemble	PlantVillage	Introduced an ensemble model for identifying tomato leaf diseases using Choquet fuzzy integrals, integrating multiple pre-trained models with fuzzy aggregation.	The aggregation method lacks flexibility and richness, with potential flaws such as not considering classifier input correlations. Also, sensitivity to outliers.
Matrouk et al. (2025)	TWDO-SAM + DCNN	Frequent Itemset Mining Dataset	Proposed a MapReduce-based framework for combining sequential association rule mining with deep learning for user categorization.	Handling large-scale datasets and efficiently processing them while maintaining model accuracy in real-time applications remains a challenge.

## 3 Methodology

**Figure 1:**
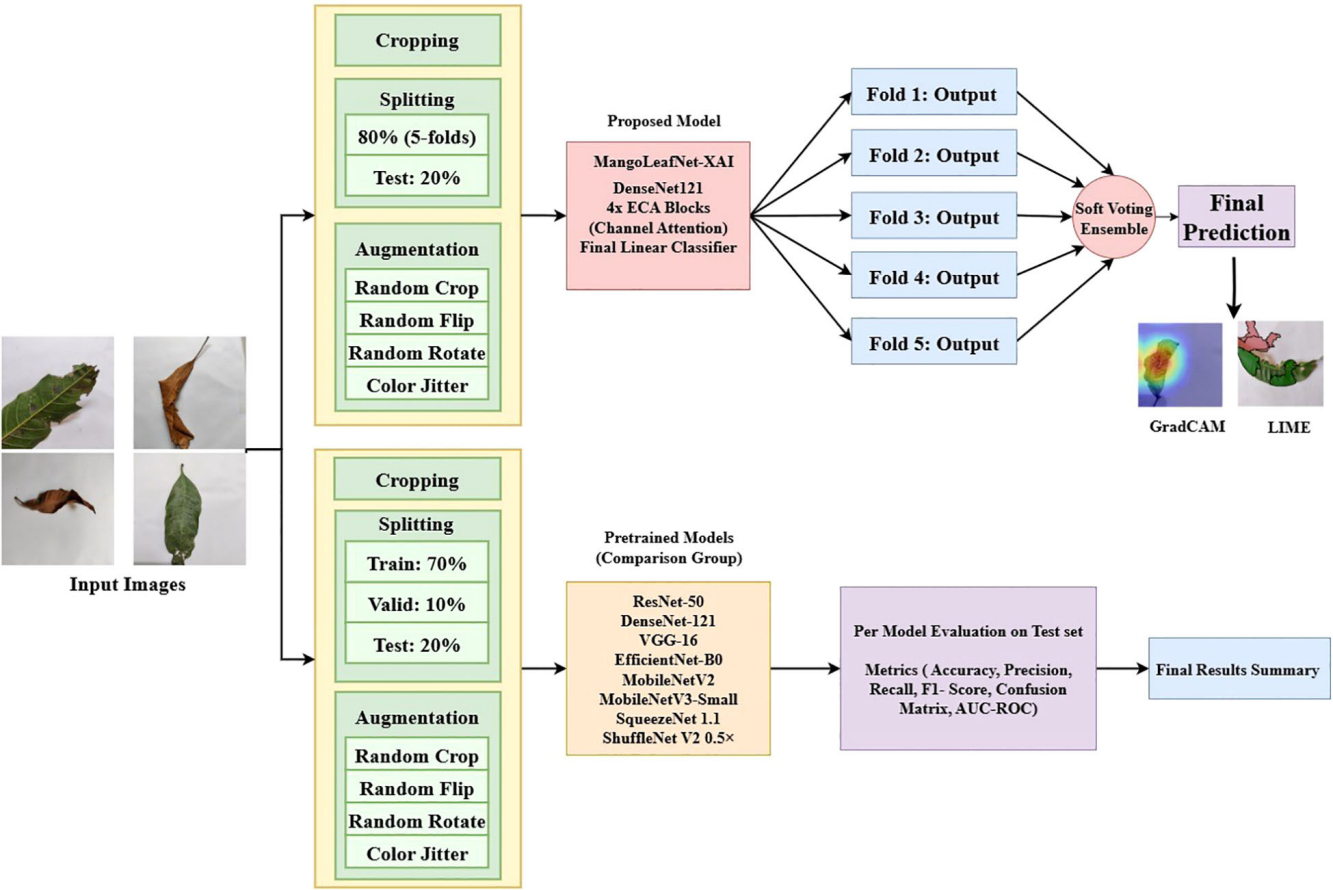
Methodology diagram. Input images adapted from Mango Leaf Disease Identification Dataset (MLDID) by Rahman Md Mizanur et al., licensed under CC BY 4.0.

This section details the comprehensive methodology for classifying mango leaf diseases, including data collection, preprocessing methods, the suggested MangoLeafNet-XAI architecture, as well as the experimental configuration. **Figure 1** provides a graphic representation of the whole process, from data collection to the ultimate forecast. To guarantee robustness and generalizability, the suggested MangoLeafNet-XAI model was thoroughly trained and assessed on three publicly accessible datasets: the Harumanis Mango Leaves Dataset 2021 (3 classes) (Gining et al., 2021), the Mango Leaf Disease Identification Dataset (MLDID) (3,000 images, 5 classes) (Rahman et al., 2024a), and the Mango Leaf Disease dataset (2,336 images, 3 classes) (Rahman et al., 2024b). Using stratified sampling, each dataset was split into a training/validation set (80%) and a distinct hold-out test set (20%). Each dataset’s training part was subjected to a 5-fold cross-validation technique. Every input image’s pixel size was adjusted to 224 x 224. In order to improve generalization and diversify the data, a variety of data augmentation methods were used during training, such as random rotations, random resizing and cropping, horizontal as well as vertical flips, and color jittering. Only scaling and normalization were applied to testing and validation sets to ensure the performance metrics reflect the model’s efficacy on real-world data distributions without artificial spatial distortions.

An already trained DenseNet-121 backbone (Huang et al., 2017) and Efficient Channel Attention (ECA) (Wang et al., 2020) modules are used together to construct the proposed model, MangoLeafNet-XAI, a deep convolutional neural network. In order to enable the model to focus on the most discriminative pathological patterns, an ECA block is inserted between each of the four DenseBlocks in order to adaptively refine features channel-wise before downsampling. In order to reduce the imbalance of classes, the Focal Loss function is applied to optimize the model. A final inference methodology is adopted to enhance stability and resilience through ensemble averaging. This method combines the predicted class probability distributions from five distinct models, with each model trained on a different fold of the five-fold cross-validation, using a soft-voting mechanism. The accuracy levels of 98.83% on MLDID, 98.09% on Mango Leaf Disease Dataset, and 98.76% on Harumanis Mango Leaves Dataset were achieved using this approach. Finally, XAI-based visualizations from Grad-CAM and LIME are employed to ensure the model’s decision-making process is grounded in verifiable pathological markers, such as necrotic spots and chlorotic lesions, rather than spurious background patterns.

### 3.1 Data acquisition & pre-processing

The proposed MangoLeafNet-XAI model has been carefully tested in relation to three mango leaf datasets to ensure that the functionality of the model and its applicability in other farming contexts have been well tested.

#### 3.1.1 Mango leaf disease identification dataset

The Mango Leaf Disease Identification Dataset (MLDID) includes a balanced data set of 3,000 images that were collected in Bangladesh. It has the same proportion for five distinct classes: Anthracnose, Bacterial Canker, Die Back, Gall Midge, and Healthy, with 600 images for each one. Images were resized and normalized to 224×224 pixels. The dataset was split with an 80–20 ratio to create a cross-validation set and a final hold-out test set. To improve the model’s generalization capability and avoid overfitting, the training data was augmented extensively with random resizing and cropping, vertical and horizontal flip, random rotation, and color jittering.

#### 3.1.2 Mango leaf disease dataset

This dataset contains 2,336 images of mango leaves collected from two orchards in Bangladesh. It is an imbalanced dataset with eight classes: Anthracnose, Bacterial Canker, Cutting Weevil, Die Back, Gall Midge, Powdery Mildew, Sooty Mould, and Healthy. As with the other datasets, images were all normalized and resized to 224×224 pixels. An 80–20 ratio was utilized for the cross-validation and hold-out test sets. A large set of data augmentation techniques, such as random resized cropping, horizontal and vertical flipping, random rotation, and color jittering, was used on the training set to enhance data variation.

#### 3.1.3 Harumanis mango leaves dataset

The Harumanis Mango Leaves Dataset contains 1,206 images of mango leaves collected from various orchards located in Perlis, Malaysia. The dataset is divided into three classes: Anthracnose, Black Sooty Mold, and Healthy. Specifically, the dataset exhibits a significant class imbalance with 525 images of Anthracnose, 457 images of Black Sooty Mold, and 224 images of Healthy leaves. This results in an imbalance ratio of approximately 2.34:1 between the majority (Anthracnose) and minority (Healthy) classes. All the images were processed uniformly by normalizing and resizing to 224×224 pixels. For model training and testing, the dataset was divided into an 80% cross-validation set and a 20% hold-out test set. Data augmentation, including random resized cropping, flipping, rotations, and color adjustment, was applied to the training images to prevent overfitting and increase robustness.

All three datasets went through the same pipeline to ensure that an appropriate and fair comparison of model performance can be made. The images were resized and normalized to 224×224 pixels, the input size the model backbones accept. Furthermore, the identical data augmentation techniques were applied to the train set of all datasets. This uniform approach helped the models learn robust and transferable features for classifying mango leaf disease. A stratified 80–20 split was consistently used across all datasets to create the primary training/validation set and a final hold-out test set, with the 80% portion being used for 5-fold cross-validation during the training phase.

## 3.2 Data augmentation, preprocessing, and experimental design

A meticulous and systematic dataset splitting process, augmentation, and preprocessing ensured the robust model evaluation and correct analysis of the comparison. To satisfy the diverse optimization requirements and functional characteristics of the pre-trained CNN architectures, the proposed MangoLeafNet-XAI experimental design incorporates techniques specific to each model. The dataset partitioning schemes used for the comparative experiments are summarized in **Table 2**.

**Table 2:** Dataset partitioning schemes for comparative experiments.

Dataset	Total images	Classes	Pre-trained models	Proposed MangoLeafNet-XAI			
			Training	Validation	Test	Training (CV)	Test
**MLDID**	3,000	5	2,100 (70%)	300 (10%)	600 (20%)	2,400 (80%)	600 (20%)
**Mango Leaf Disease**	2,353	8	1,647 (70%)	235 (10%)	471 (20%)	1,882 (80%)	471 (20%)
**Harumanis**	1,206	3	844 (70%)	120 (10%)	242 (20%)	964 (80%)	242 (20%)

The parameterization and intended generalization error serve as a guidance for the minimal training set size for pre-trained architectures:

(1) $$n_{\text{train}}\geq \frac{p\cdot d}{\epsilon^{2}}$$

In Equation 1, d is the input dimensionality, ϵ is the permitted generalization error, and p is the total number of trainable parameters.

The estimated predicted generalization error for MangoLeafNet-XAI using k-fold cross-validation is as follows:

(2) $$E\left[L_{\text{CV}}]=E[L_{\text{true}}\right]+O\left(\frac{1}{k}\right)+O\left(\sqrt{\frac{d}{n}}\right)$$

In order to properly balance bias and variance, using k=5 folds, as defined in Equation 2.

To match the input distributions of the pre-trained model, all datasets were normalized. ImageNet statistics were used for normalization:


$$x_{\text{normalized}}=\frac{x-\mu }{\sigma },\;\mu =\left[0.485,0.456,0.406],\;\sigma =[0.229,0.224,0.225\right]$$

guaranteeing transfer learning weight compatibility and promoting steady gradient propagation.

To preserve pathological semantics, expand the effective dataset size, and strengthen model resilience, a comprehensive augmentation procedure was implemented. The specific augmentation transformations and their underlying rationale are detailed in **Table 3**.

**Table 3:** Augmentation transformations and rationale.

Transformation	Parameters	Mathematical formulation	Biological rationale
RandomResizedCrop	size=224, scale=(0.7,1.0)	$x^{\prime }=\text{Crop}\left(\text{RandomScale}\left(x\right)\right)$	Simulates variable imaging distances
RandomHorizontalFlip	p=0.5	$x^{\prime }={\text{Flip}}_{h}\left(x\right)$w.p. 0.5	Accounts for leaf orientation invariance
RandomVerticalFlip	p=0.5	$x^{\prime }={\text{Flip}}_{v}\left(x\right)$w.p. 0.5	Captures presentation variability
RandomRotation	± 30°	$x^{\prime }={\text{Rotate}}_{\theta }\left(x\right),\theta ∼U\left(-30{}^{\circ},30{}^{\circ}\right)$	Accommodates natural leaf rotations
ColorJitter	brightness=0.2–0.3, contrast=0.2–0.3	$x^{\prime }={\text{Jitter}}_{color}\left(x\right)$	Compensates for illumination variations
Normalization	ImageNet statistics	$x_{\text{norm}}=\left(x-\mu \right)/\sigma$	Standardizes input distribution

### 3.3 Proposed MangoLeafNet-XAI architecture

The proposed MangoLeafNet-XAI is a meticulously designed, innovative deep learning framework of deep learning that may be applied in the extremely precise classification of mango leaf diseases. Unlike the traditional CNN models, MangoLeafNet-XAI has a hybrid paradigm, which synthetically couples with jointly the Efficient Channel Attention (ECA) modules, and a DenseNet-121 backbone mutually to improve its discriminative capacity through adaptive channel-wise feature recalibration.

#### 3.3.1 Architectural overview

**Figure 2:**
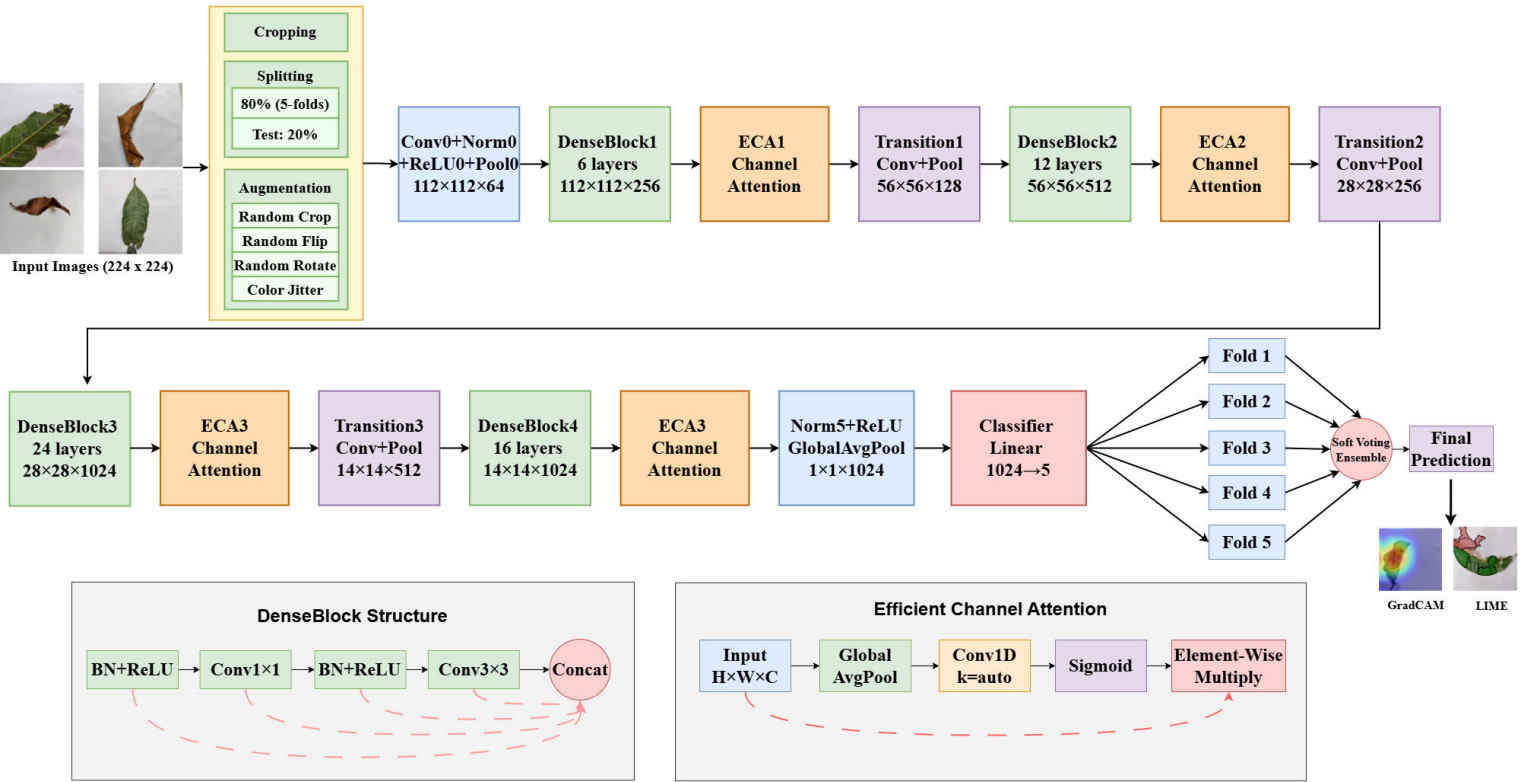
Input images adapted from Mango Leaf Disease Identification Dataset (MLDID) by Rahman Md Mizanur et al., licensed under CC BY 4.0.

The main feature extractor used by MangoLeafNet-XAI is a pre-trained DenseNet-121 model, which takes use of its dense connection to facilitate effective gradient propagation and feature reuse. By enabling strong low- and mid-level feature transfer, the pre-trained weights derived from ImageNet shorten convergence times and enhance generalization. The proposed MangoLeafNet-XAI architecture illustrating this design is presented in **Figure 2**.

ECA modules are inserted sequentially right after each DenseBlock, which is the main novelty of MangoLeafNet-XAI. The output of the DenseBlock passes exclusively through the ECA module for recalibration before entering the subsequent Transition layer. Through adaptive learning of channel interdependencies, these modules improve the intermediate feature representations, suppressing redundant or noisy activations and amplifying relevant pathogenic signals.

The ECA module formally uses Global Average Pooling (GAP) across the spatial dimensions to provide a compact channel descriptor given an input feature map X∈R^(H×W×C)^ from a DenseBlock:

(3) $$z_{c}=\frac{1}{H\times W}\sum_{i=1}^{H}\sum_{j=1}^{W}X_{c}\left(i,j\right),\;z\in {\mathbb{R}}^{C}$$

Where ***H*** and ***W*** represent the spatial height and width, ***C*** denotes the number of channels, and ***X_c_ (i,j)*** refers to the pixel value at spatial coordinates for the ***c***-th channel, as defined in Equation 3.

In order to efficiently simulate local cross-channel interactions with no dimensionality reduction, this descriptor is then processed using a 1D convolution with an adaptively chosen kernel size *k*:


a=σ(Conv1D(z;k))

Where, $a\in {\mathbb{R}}^{C}$ stands for the attention weights and σ(·) for the sigmoid activation function. After recalibration, the final feature map $\hat{X}$ is as follows:


$${\hat{X}}_{c}=a_{c}\cdot X_{c},\;c=1,2,\ldots ,C$$

Through the use of this dynamic scaling technique, MangoLeafNet-XAI’s classification resilience is strengthened by maintaining a fine-grained focus on disease-relevant channels.

#### 3.3.2 Classification head

Global Average Pooling (GAP) is applied to the final feature mappings $X_{4}\in {\mathbb{R}}^{7\times 7\times 1024}$ generated by the last ECA module, producing a feature vector of 1024 dimensions, as defined in Equation 4:

(4) $$f=\frac{1}{H\times W}\sum_{i=1}^{H}\sum_{j=1}^{W}X_{4}\left(i,j\right)$$

This vector undergoes a ReLU activation to add non-linearity after being normalized using Batch Normalization (BN), as defined in Equation 5:

(5) $$h=\sigma_{\text{ReLU}}\left(\text{BN}\left(f\right)\right)$$

In order to determine class probabilities, a softmax activation is performed after a fully connected layer maps h into the output space of N illness classes, as defined in Equation 6:

(6) $$y=\text{softmax}\left(Wh+b\right),\;y\in {\mathbb{R}}^{N}$$

$W\in {\mathbb{R}}^{N\times 1024}\;$and $b\in {\mathbb{R}}^{N}$ represent the classification layer’s trainable parameters, respectively.

#### 3.3.3 Key architectural components

Feature Extraction Backbone DenseNet-121, which mitigates vanishing gradient problems by guaranteeing significant feature reuse via dense connectedness.

Integration of Attention Adaptive channel re-weighting is made possible by four ECA modules placed after DenseBlocks.

The classification head which offers a condensed but effective conversion from the feature space to class probabilities, is GAP → BN → ReLU → Fully Connected → Softmax.

#### 3.3.4 Optimization strategy

In order to address class imbalance, MangoLeafNet-XAI uses the Focal Loss function [γ=2.0,α=0.25], which highlights difficult-to-classify data and downweights simple examples:

(7) $${ℒ}_{\text{focal}}\left(p_{t}\right)=-\alpha {\left(1-p_{t}\right)}^{\gamma }\text{log}\left(p_{t}\right)$$

In Equation 7, $p_{t}$ represents the expected probability associated with the ground truth class.

The AdamW optimizer is used to improve the network, with a weight decay of $1\times 10^{-3}$ and an initial learning rate of $1\times 10^{-4}$. A ReduceLROnPlateau scheduler is used to guarantee adaptive convergence.

#### 3.3.5 Parameter efficiency

With only 6.9 million trainable parameters, far fewer than many state-of-the-art CNN variants, MangoLeafNet-XAI achieves an effective balance between performance and complexity while maintaining excellent classification accuracy. The layer-wise architecture of MangoLeafNet-XAI is presented in **Table 4**. While lightweight models such as MobileNetV2 and EfficientNet-B0 have smaller parameter footprints, they often fail to capture the subtle, fine-grained morphological features of mango diseases, leading to an accuracy drop of nine to eleven percent in our experiments. MangoLeafNet-XAI justifies its 6.9 million parameters by achieving a substantial performance gain with 98.83% accuracy while remaining well below the ten million parameter limit typically required for efficient deployment on edge devices such as Raspberry Pi or mobile-based agricultural scanners. This offers a superior trade-off for high-stakes disease diagnosis, where precision is as important as efficiency.

**Table 4:** Layer-wise architecture of MangoLeafNet-XAI.

Layer type	Output size	Kernel size/stride	Parameters	Notes
Input	224×224×3	–	–	RGB image
Initial Conv	112×112×64	7×7/2	9,408	BN + ReLU
Max Pool	56×56×64	3×3/2	–	–
DenseBlock 1	56×56×256	[1×1, 3×3]×6	200,704	Growth=32
ECA Module 1	56×56×256	Adaptive	–	Channel attention
Transition 1	28×28×128	1×1 + 2×2 pool	33,024	θ=0.5
DenseBlock 2	28×28×512	[1×1, 3×3]×12	535,808	Growth=32
ECA Module 2	28×28×512	Adaptive	–	Channel attention
Transition 2	14×14×256	1×1 + 2×2 pool	132,096	θ=0.5
DenseBlock 3	14×14×1024	[1×1, 3×3]×24	1,576,704	Growth=32
ECA Module 3	14×14×1024	Adaptive	–	Channel attention
Transition 3	7×7×512	1×1 + 2×2 pool	525,312	θ=0.5
DenseBlock 4	7×7×1024	[1×1, 3×3]×16	1,357,824	Growth=32
ECA Module 4	7×7×1024	Adaptive	–	Channel attention
Classification				
GAP	1×1×1024	7×7	–	Feature aggregation
Batch Norm	1×1×1024	–	2,048	Regularization
ReLU	1×1×1024	–	–	Non-linearity
Fully Connected	1×1×N	–	N×1024	Class projection
Softmax	1×1×N	–	–	Probability distribution

Total Parameters: 6,903,104+(N×1024).

## 3.4 Comparative analysis of pre-trained architectures for mango leaf disease classification

A thorough comparison of eight cutting-edge pre-trained convolutional neural network (CNN) architectures for the classification of mango leaf disease is presented in this part. Both deep designs with better representational learning capabilities and lightweight architectures intended for deployment in resource-constrained situations are taken into consideration in the study.

### 3.4.1 Experimental framework

All architectures undergo training using a same experimental approach to provide an equitable comparison. In order to implement transfer learning, a fully linked layer appropriate for five-class classification is used in place of the original classification head. A 70-10–20 stratified split is used to separate the dataset into training, validation, and testing subsets while maintaining the class distribution.

For better generality, optimization uses the AdamW method, which incorporates weight decay into Adam. The definition of the update rule is:

(8) $$\theta_{t+1}=\theta_{t}-\eta \cdot \frac{{\hat{m}}_{t}}{\sqrt{{\hat{v}}_{t}}+\epsilon }-\lambda \theta_{t}$$

In Equation 8, η stands for the learning rate ( $3\times 10^{-6}$), $\lambda =1\times 10^{-4}$ for the weight decay, ${\hat{m}}_{t}$ and $\hat{v}$ for bias-corrected first and second moment estimations, and ϵ is a tiny coefficient for numerical stability.

Early stopping with a 5-epoch patience halts training when validation performance stagnates, whereas ReduceLROnPlateau scheduling dynamically modifies the learning rate to further improve convergence and reduce overfitting.

### 3.4.2 Architectural overview

From dense and residual connection to very effective depthwise separable convolutions, the assessed models cover a wide range of architectural philosophies:

ResNet-50 uses identity-based skip connections to overcome vanishing gradients by introducing residual learning:

(9) $$y=ℱ\left(x,\left\{W_{i}\right\}\right)+x$$

The input identity in Equation 9 represented by x, and the residual mapping is shown by $ℱ\left(x,\left\{W_{i}\right\}\right)$. It uses bottleneck blocks to increase computing performance while maintaining depth in its 50 layers.

DenseNet-121 uses dense connection, in which all inputs from previous levels are received by each layer, as shown in Equation 10:

(10) $$x_{\ell }=H_{\ell }\left(\left[x_{0},x_{1},\ldots ,x_{\ell -1}\right]\right)$$

Reducing disappearing gradients and allowing feature reuse. DenseNet outperforms VGG-like architectures in terms of parameter efficiency and compactness because of its 121 layers.

VGG-16 uses only tiny 3x3 kernels and has a uniform architecture with 16 weight layers. Its simple design provides deep feature hierarchies, despite the computational cost (138M parameters).

EffectiveNet-B0 Compound scaling across depth (d), width (w), and resolution (r) is introduced by EfficientNet and is described as follows:


$$d=\alpha^{\phi },\;w=\beta^{\phi },\;r=\gamma^{\phi }$$

Considering the restriction $\alpha \cdot \beta^{2}\cdot \gamma^{2}\approx 2$. Neural architecture search (NAS) was used to improve EfficientNet-B0, the baseline, for a balance between accuracy and efficiency.

Different MobileNet Versions (v2, v3-small) Inverted residuals exhibiting linear bottlenecks are used in MobileNets, as shown in Equation 11:

(11) $$y={\text{Conv}}_{1\times 1}\left({\text{DWConv}}_{k\times k}\left({\text{Conv}}_{1\times 1}\left(x\right)\right)\right)$$

In this case, DWConv represents depthwise convolutions, while Conv denotes pointwise ${\text{Conv}}_{1\times 1}$. NAS-based improvements are included into MobileNetV3 for deployment that is hardware-aware.

SqueezeNet version 1.1 SqueezeNet uses fire modules, which include expand layers (1×1 along with 3×3 filters) after a squeeze layer (1×1 filters) decreases channel size. With only 1.2M parameters, this produces very high parameter efficiency.

ShuffleNet V2 (0.5x) For effective representation, ShuffleNet uses group convolutions and channel shuffle:


y=Shuffle(GroupConv(x))

Consequently, low computational cost and precision are balanced for mobile devices.

### 3.4.3 Classification mechanism

The categorization framework is the same for all architectures. A linear layer and softmax are used to transfer the penultimate feature representation $h\left(x\right)\in {\mathbb{R}}^{d}$to class probabilities:

(12) $$p(y=k|x)=\frac{\text{exp}\left(W_{k}^{⊤}h\left(x\right)+b_{k}\right)}{\sum_{j=1}^{K}\text{exp}\left(W_{j}^{⊤}h\left(x\right)+b_{j}\right)}$$

In Equation 12, $b\in {\mathbb{R}}^{K}$ and, $W\in {\mathbb{R}}^{d\times K}$. This guarantees that the five illness categories will be interpreted probabilistically.

Reducing cross-entropy loss across N samples and K classes is the learning objective:

(13) $$ℒ=-\sum_{i=1}^{N}\sum_{j=1}^{K}y_{ij}\text{log}\left({\hat{y}}_{ij}\right)$$

In Equation 13, $y_{ij}$ represents the one-hot ground truth, and ${\hat{y}}_{ij}$ represents the class j projected probability.

ImageNet statistics are used to normalize the input images (μ=[0.485,0.456,0.406], σ=[0.229,0.224,0.225]) and scale them to 224×224 pixels. Data augmentation uses color jittering, ± 30° rotation, horizontal/vertical flipping, and random resizing cropping (0.8-1.0 scale) to enhance generalization. A batch size of 32 is used for training in order to balance computational viability with consistent gradient estimation.

### 3.4.4 Theoretical considerations

The research also examines the trade-off between bias and variance in various topologies. While lightweight models (MobileNets, SqueezeNet) often incur more bias but generalize better with limited data, deep models (ResNet-50, DenseNet-121) show lower bias but larger variance.

According to Neural Tangent Kernel (NTK) theory, enhanced fine-grained illness identification is made possible because deeper networks converge to kernels exhibiting richer feature representations. Comparative evaluation remains essential, although it leads to higher computational costs. The architectural specifications and theoretical parameter counts that support this comparison are detailed in **Table 5**.

**Table 5:** Architectural specifications and theoretical parameter counts.

Model	Depth	Parameters (M)	Theoretical framework	Special characteristics
ResNet-50	50	25.6	Residual Learning	Identity mapping, bottleneck blocks
DenseNet-121	121	8.1	Dense Connectivity	Feature reuse, concatenation
VGG-16	16	138	Homogeneous Design	Small receptive fields
EfficientNet-B0	–	5.3	Compound Scaling	NAS-optimized scaling
MobileNetV2	53	3.5	Inverted Residuals	Linear bottlenecks
MobileNetV3-Small	–	2.5	NAS Optimization	Hardware-aware efficiency
SqueezeNet 1.1	18	1.2	Fire Modules	Extreme parameter efficiency
ShuffleNet V2 0.5×	–	1.4	Channel Shuffle	Group convolutions

### 3.5 Loss function and ensemble strategy

We used the Focal Loss to counteract the negative impacts of class imbalance and the propensity of traditional cross-entropy loss to be dominated by well-classified data. In contrast to categorical cross-entropy, which penalizes all misclassifications equally, Focal Loss dynamically reweights each training example’s contribution according to how difficult it is to classify. The Focal Loss is defined formally as follows, assuming the expected probability for the true class $p_{t}$:

(14) $${ℒ}_{\text{FL}}\left(p_{t}\right)=-\;\alpha \left(1-p_{t}\right)\gamma \text{log}\left(p_{t}\right)$$

In Equation 14, $p_{t}=\frac{\text{exp}\left(z_{y}\right)}{\sum_{c=1}^{C}\text{exp}\left(z_{c}\right)}$, where, $z_{y}$ is the logit associated with the ground-truth class *y* among C classes. The degree of attention on misclassified samples and the balance between classes are controlled by the parameters α∈(0,1) and γ≥0, respectively. The loss reduces to the standard cross-entropy formulation at γ = 0.

In our approach, we used α=0.25 and γ=2.0 to make categories of difficult-to-classify illness contribute more towards the optimization process. By preserving overall stability during training, this framework improved the discriminative capability of the network and led to better sensitivity towards minority classes.

To improve generality and resilience, we used a K-fold stratified ensemble approach. To ensure uniformity in the distribution of classes across partitions, the training data were specifically divided into K = 5 folds. The remaining folds were utilized as training data, and each fold was used once as a validation set. The ensemble was created by combining the prediction distributions derived from each fold-specific model.

For an input picture x, let ${\hat{p}}_{k}\left(x\right)\in {\mathbb{R}}^{C}$represent the projected probability vector derived from the model trained on the k^th fold. The arithmetic mean across all folds provides the final ensemble prediction, as shown in Equation 15:

(15) $${\hat{p}}_{\text{ens}}\left(x\right)=\frac{1}{K}\sum_{k=1}^{K}{\hat{p}}_{k}\left(x\right)$$

Next, the anticipated class label is chosen based on, as shown in Equation 16:

(16) $$\hat{y}=\text{arg}\underset{c\in \left\{1,\ldots ,C\right\}}{\text{max}}{\hat{p}}_{\text{ens},c}\left(x\right)$$

To reduce variance and overfitting, this soft-voting ensemble effectively uses additional decision limits which have been trained on multiple data subsets. The ensemble was more stable and predictive in comparison to an individual model instance. On the one hand, in applications where the datasets are sparsely distributed or unbalanced as in agricultural disease classification, such aggregation not only exploits the diversity of the individual folds but also the use of the entire training dataset.

### 3.6 Experimental setup and computational efficiency

To ensure the reproducibility of the results and evaluate the real-world deployability of the proposed architecture, all experiments were conducted within a standardized computational environment. The implementation utilized the PyTorch deep learning framework (Python 3.10) executed on a Kaggle notebook environment. The hardware specifications included an Intel(R) Xeon(R) CPU (2.00GHz, 2 physical cores, 4 logical cores), 30 GB of RAM, and an NVIDIA Tesla P100 GPU with 16 GB of HBM2 VRAM.

All models were trained using a consistent configuration to ensure a fair comparison: a batch size of 32, the AdamW optimizer with a weight decay of $1\times 10^{-3}$, and a Stratified 5-Fold Cross-Validation scheme.

#### 3.6.1 Computational complexity analysis

Beyond classification accuracy, the suitability of a model for agricultural automation depends heavily on computational efficiency and inference latency. **Table 6** details the training duration and inference speed of MangoLeafNet-XAI compared to state-of-the-art pre-trained models.

**Table 6:** Training and inference performance comparison on MLDID.

Model	Avg epoch time (sec)	Total training time (min)	Total inference time (sec)	Per sample inference (ms)	Parameters (M)
ResNet-50	13.04	3.26	2.0692	3.4487	25.6
DenseNet-121	12.80	3.20	2.1603	3.6005	8.1
VGG-16	19.64	4.91	2.2004	3.6674	138
EfficientNet-B0	11.96	2.99	2.0710	3.4516	5.3
MobileNetV2	11.52	2.88	1.9496	3.2494	3.5
MobileNetV3-Small	10.88	2.72	2.0568	3.4281	2.5
SqueezeNet 1.1	10.80	2.70	2.0069	3.3448	1.2
ShuffleNet V2 0.5×	10.96	2.74	1.9591	3.2651	1.4
MangoLeafNet-XAI	**11.36**	**2.84**	**2.0275**	**3.3792**	**6.9**

The proposed MangoLeafNet-XAI demonstrates a highly favorable trade-off between speed and performance. With an average inference time of 3.38 ms per sample, the model is capable of processing approximately 296 frames per second, making it well-suited for real-time applications. While lightweight models such as ShuffleNet V2 and SqueezeNet offer slightly lower latency (~3.27 ms and ~3.34 ms, respectively), they suffer from significant drops in accuracy. Conversely, VGG-16 requires significantly higher computational resources, with a training time of 4.91 minutes and a higher inference latency, without yielding a proportional accuracy benefit. MangoLeafNet-XAI achieves near-state-of-the-art speed while maintaining the high accuracy characteristics of deeper architectures.

## 4 Result analysis

In this section, the three benchmark datasets used in the overall test of the state-of-art pre-trained convolutional neural networks are Mango Leaf Disease Dataset, Mango Leaf Disease Identification Dataset (MLDID), and the Harumanis Mango Leaves. The results that were applied to assess the performance are commonly used that include accuracy, recall, macro-averaged precision and F1-score; weighted and micro-averages have been used, following established protocols for plant disease identification on imbalanced datasets (Salka et al., 2025). This comparative study presents the relative merits and demerits of other topologies and ways they can be applied to factual farm disease detection.

### 4.1 Quantitative assessment of the pretrain models

EfficientNetB0, ShuffleNet V2, MobileNetV3 Small, ResNet50, DenseNet121, and VGG16 were evaluated on three datasets, with results in **Table 7** showing performance differences based on dataset and architecture. On the Mango Leaf Disease Identification Dataset, deeper models demonstrated superior performance compared to lightweight architectures. VGG16 achieved 96.67% accuracy and 96.63% macro F1-score, DenseNet121 reached 94.67%, ResNet50 91.83%, while ShuffleNet V2 x0.5 only attained 52.17%, revealing limitations of lightweight networks in handling complex features.

**Table 7:** Comprehensive performance of pre-trained models – MLDID, Mango Leaf Disease, and Harumanis Mango Leaves Datasets.

Performance of pre-trained models on the MLDID dataset				
Model	Accuracy	Macro precision	Macro recall	Macro F1-score
MobileNet V2	0.8733	0.8792	0.8733	0.8684
EfficientNet-B0	0.8983	0.9037	0.8983	0.8938
SqueezeNet1_1	0.8400	0.8416	0.8400	0.8399
ShuffleNet V2 x0.5	0.5217	0.8046	0.5217	0.4833
MobileNet V3 Small	0.8083	0.8400	0.8083	0.8033
ResNet50	0.9183	0.9237	0.9183	0.9163
DenseNet121	0.9467	0.9474	0.9467	0.9461
VGG16	0.9667	0.9671	0.9667	0.9663
Performance of pre-trained models on the mango leaf disease dataset				
Model	Accuracy	Macro precision	Macro recall	Macro F1
MobileNetV2	0.9363	0.9428	0.9416	0.9421
EfficientNetB0	0.9448	0.9496	0.9523	0.9501
SqueezeNet1_1	0.1380	0.0173	0.1250	0.0303
ShuffleNetV2	0.9618	0.9631	0.9672	0.9647
MobileNetV3	0.7028	0.8142	0.6648	0.6516
ResNet50	0.7622	0.8004	0.7501	0.7583
DenseNet121	0.7580	0.7680	0.7573	0.7465
VGG16	0.1529	0.0191	0.1250	0.0331
Performance of pre-trained models on the harumanis mango leaves dataset				
Model	Accuracy	Macro precision	Macro recall	Macro F1
MobileNet_V2	0.7603	0.7663	0.7109	0.7257
EfficientNet_B0	0.8140	0.8163	0.7203	0.7241
SqueezeNet1_1	0.8843	0.8709	0.9044	0.8812
ShuffleNet_V2_X0_5	0.5661	0.5791	0.5144	0.5214
MobileNet_V3_Small	0.6612	0.6253	0.5750	0.5668
ResNet50	0.9050	0.9061	0.9065	0.9062
DenseNet121	0.9298	0.9420	0.9376	0.9364
VGG16	0.9587	0.9576	0.9504	0.9538

The empirical evaluation is conducted on MangoLeafNet-XAI, a DenseNet-121 backbone augmented with Efficient Channel Attention (ECA) modules and optimized through the application of Focal Loss. The model was rigorously assessed on three heterogeneous mango leaf disease datasets- MLDID, Mango Leaf Disease Dataset, and Harumanis Mango Leaves- using 5-fold cross-validation and ensemble aggregation to ensure statistical robustness and generalizability.

Performance on the Mango Leaf Disease Dataset Significantly distinct patterns were seen in the Mango Leaf Disease dataset. ShuffleNet V2 (x1.0) achieved a macro F1-score of 96.47% and an accuracy of 96.18%, outperforming all other models. MobileNetV2 (93.63%) and EfficientNetB0 (94.48%) both demonstrated excellent performance. With accuracies of 15.29 percent and 13.80 percent respectively, VGG16 and SqueezeNet1_1 exhibited near-random performance. We attribute VGG16’s failure to its excessive parameter density (138M), which likely led to severe overfitting on the smaller, highly variable Mango Leaf Disease dataset. Conversely, SqueezeNet’s failure highlights the limitations of extreme compression; its limited capacity was insufficient to capture the fine-grained texture differences between ‘Sooty Mould’ and ‘Black Sooty Mold’ under varying lighting conditions. The poor performance of VGG16 and SqueezeNet1_1 on the eight-class Mango Leaf Disease dataset, achieving only around fourteen to fifteen percent accuracy, indicates a failure in gradient propagation or an inability to capture fine-grained features in the presence of high class imbalance and background noise. Lightweight models such as ShuffleNet V2 exhibited high variance across datasets, suggesting that their limited parameter capacity is insufficient to generalize across diverse geographic regions and illumination conditions. MangoLeafNet-XAI demonstrates superior stability across all three datasets due to the ECA modules, which refine features channel-wise. This enables the model to emphasize pathogenic signals, such as the necrotic edges of Anthracnose, while suppressing irrelevant background noise that caused the collapse of traditional architectures.

Performance on the Harumanis Mango Leaves Dataset Deeper structures were again shown to be better on the Harumanis dataset. The best accuracy (95.87%) and weighted F1-score (95.85%) were obtained by VGG16, which was followed by DenseNet121 (92.98%) and ResNet50 (90.50%). Remarkably, SqueezeNet1_1 did well here (88.43%) despite failing on the Mango Leaf Disease dataset. With accuracies of 66.12 percent and 56.61 percent respectively, lightweight models such as MobileNetV3 Small and ShuffleNet V2 x0.5 again delivered weak performance.

### 4.2 Quantitative assessment of the proposed model

#### 4.2.1 Mango leaf disease identification dataset

**Figure 3:**
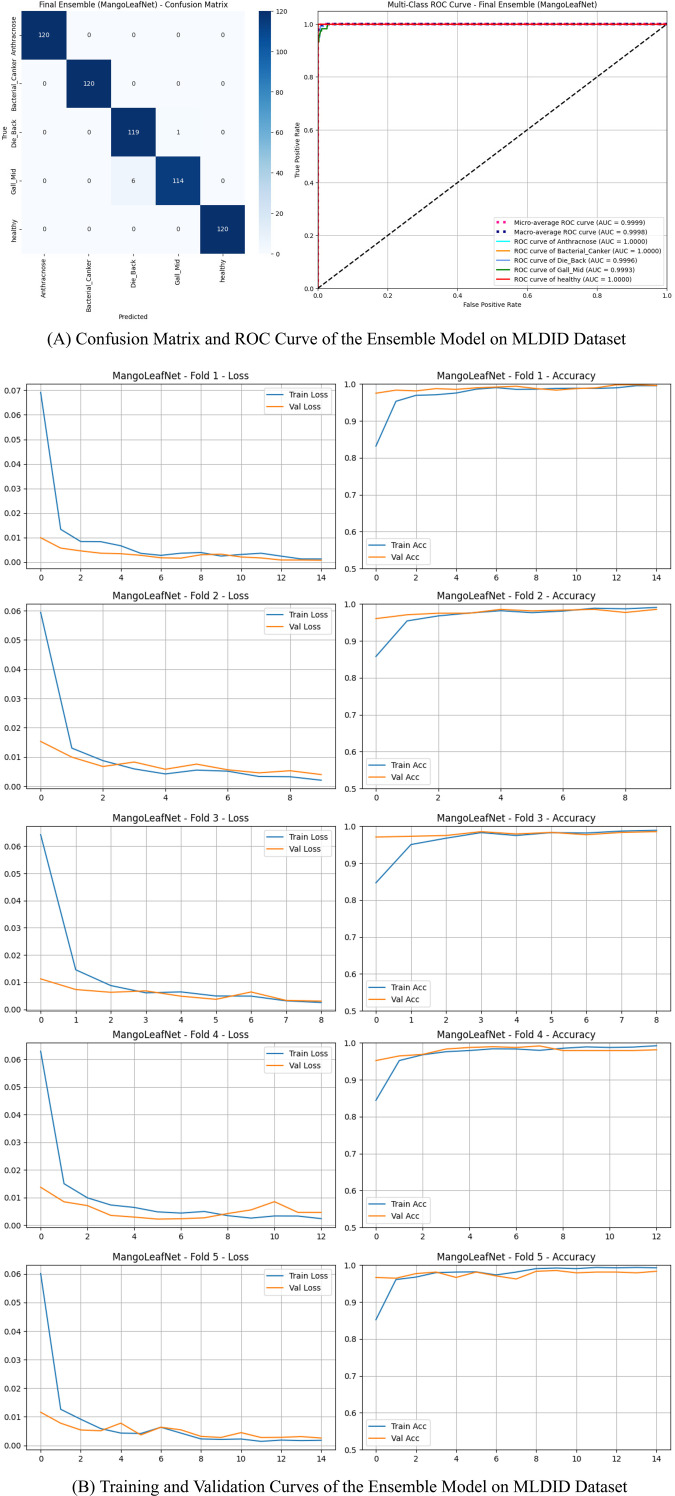
Comprehensive performance visualization of the ensemble model on the MLDID dataset. **(A)** Confusion Matrix and ROC Curve of the Ensemble Model on MLDID Dataset. **(B)** Training and Validation Curves of the Ensemble Model on MLDID Dataset.

There are five classifications in the dataset: Healthy, Die Back, Bacterial Canker, Anthracnose, and Gall Midge. **Table 8** provides a summary of the cross-validation and ensemble outcomes. Further insights into the model’s behavior are illustrated in **Figure 3**, which presents the confusion matrix and ROC curve of the ensemble model on the MLDID dataset. The confusion matrix in **Figure 3A** shows that most classes achieved near-perfect scores, but there is slight confusion between Gall Midge and Die Back. This confusion is attributed to the similar early-stage lesion morphology characterized by small brown spots, which even deeper models such as ResNet50 found difficult to differentiate. Nonetheless, the ensemble averaging in our model effectively reduces errors from individual folds, leading to a final state-of-the-art accuracy of 98.83%.

**Table 8:** Comprehensive 5-fold cross-validation results - MLDID, mango leaf disease, and harumanis datasets.

5-fold cross-validation results on the MLDID dataset				
Fold	Accuracy	Macro precision	Macro recall	Macro F1-Score
1	0.9917	0.9917	0.9917	0.9917
2	0.9817	0.9818	0.9817	0.9816
3	0.9767	0.9774	0.9767	0.9767
4	0.9850	0.9852	0.9850	0.9850
5	0.9933	0.9934	0.9933	0.9933
Mean	0.9857	0.9859	0.9857	0.9857
5-fold cross-validation results on the mango leaf disease dataset				
Fold	Accuracy	Macro precision	Macro recall	Macro F1-Score
1	0.9660	0.9688	0.9683	0.9683
2	0.9809	0.9825	0.9828	0.9826
3	0.9575	0.9590	0.9609	0.9593
4	0.9724	0.9756	0.9753	0.9748
5	0.9766	0.9783	0.9776	0.9778
Mean	0.9707	0.9728	0.9730	0.9726
5-fold cross-validation results on harumanis dataset				
Fold	Accuracy	Macro precision	Macro recall	Macro F1-Score
1	0.9793	0.9753	0.9841	0.9793
2	0.9835	0.9760	0.9864	0.9808
3	0.9835	0.9823	0.9869	0.9845
4	0.9793	0.9757	0.9832	0.9793
5	0.9711	0.9685	0.9773	0.9723
Mean	0.9793	0.9756	0.9836	0.9793

High model stability was shown by the closely matched metrics across folds and the mean accuracy of 98.57%. **Table 9** presents the comprehensive performance of the final ensemble model across the MLDID, Mango Leaf Disease, and Harumanis datasets, summarizing its effectiveness and consistency under diverse classification settings.

**Table 9:** Comprehensive final ensemble model performance - MLDID, Mango Leaf Disease, and Harumanis Datasets.

Final ensemble model performance on the MLDID dataset			
Class	Precision	Recall	F1-Score
Anthracnose	1.0000	1.0000	1.0000
Bacterial Canker	1.0000	1.0000	1.0000
Die Back	0.9520	0.9917	0.9714
Gall Midge	0.9913	0.9500	0.9702
Healthy	1.0000	1.0000	1.0000
Accuracy			0.9883
Macro Avg	0.9887	0.9883	0.9883
Weighted Avg	0.9887	0.9883	0.9883
Final ensemble model performance on the mango leaf disease dataset			
Class	Precision	Recall	F1-Score
Anthracnose	0.9841	0.9538	0.9688
Bacterial Canker	0.9718	0.9583	0.9650
Cutting Weevil	0.9688	1.0000	0.9841
Die Back	1.0000	1.0000	1.0000
Gall Midge	0.9683	0.9531	0.9606
Healthy	1.0000	1.0000	1.0000
Powdery Mildew	1.0000	1.0000	1.0000
Sooty Mould	0.9636	1.0000	0.9815
Accuracy			0.9809
Macro Avg	0.9821	0.9832	0.9825
Weighted Avg	0.9810	0.9809	0.9808
Final model performance on harumanis dataset			
Class	Precision	Recall	F1-Score
Anthracnose	1.0000	0.9714	0.9855
Black Sooty Mold	0.9892	1.0000	0.9946
Healthy	0.9574	1.0000	0.9783
Accuracy			0.9876
Macro Avg	0.9822	0.9905	0.9861
Weighted Avg	0.9880	0.9876	0.9876

Three classes had perfect categorization, and the ensemble’s accuracy was 98.83%. There was some little misunderstanding between the conditions Gall Midge and Die Back, which have similar visual characteristics.

#### 4.2.2 Mango leaf disease dataset

**Figure 4:**
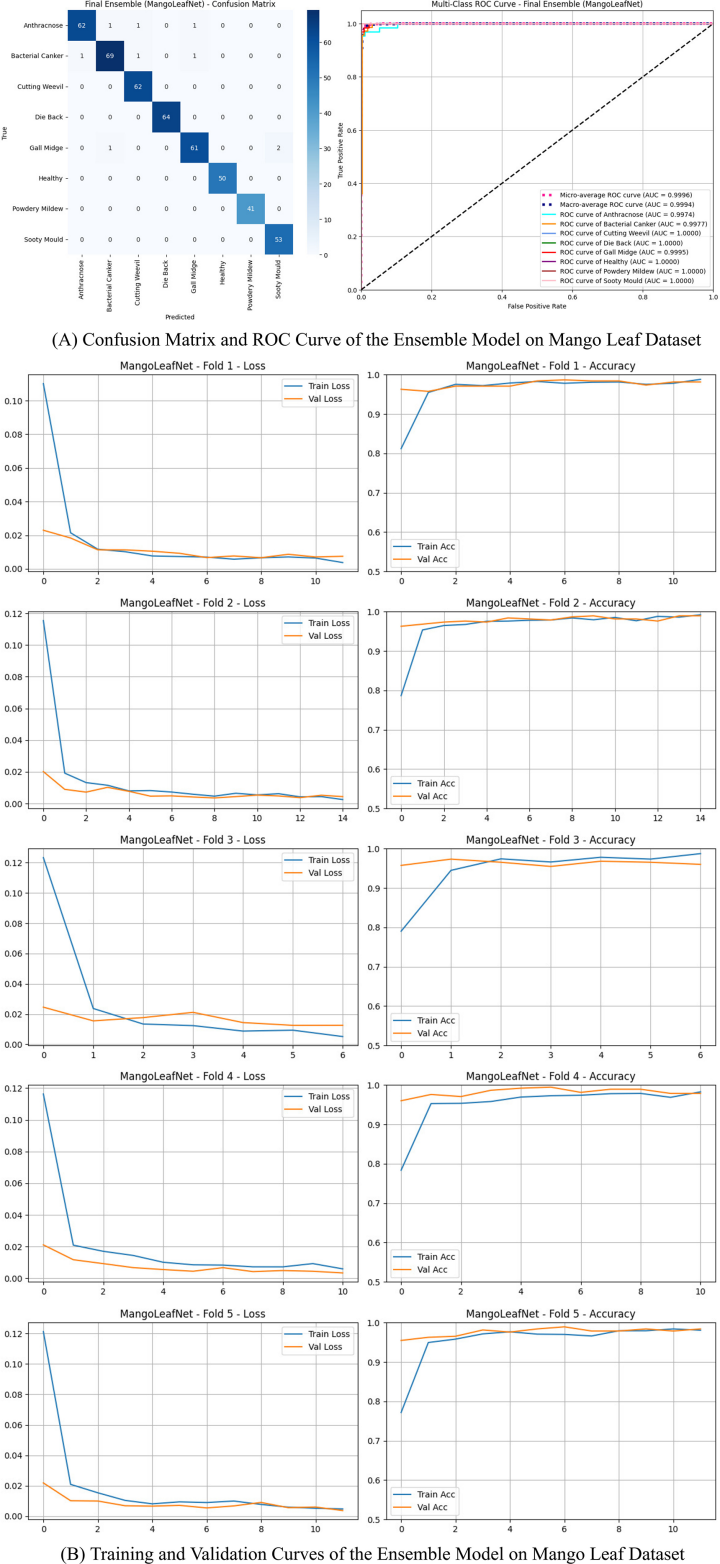
Comprehensive performance visualization – ensemble model (mango leaf dataset). **(A)** Confusion Matrix and ROC Curve - Ensemble Model (Mango Leaf Dataset). **(B)** Training and Validation Curves - Ensemble Model (Mango Leaf Dataset).

This dataset contains eight classifications that include seven disease categories and one healthy class. While the performance of the model is further represented in **Figure 4** for the confusion matrix and ROC curve for the training and validation curves of the Mango Leaf Dataset.

The group’s accuracy was 98.09%, and Die Back, Healthy, and Powdery Mildew were all perfectly recognized.

#### 4.2.3 Harumanis mango leaves dataset

**Figure 5:**
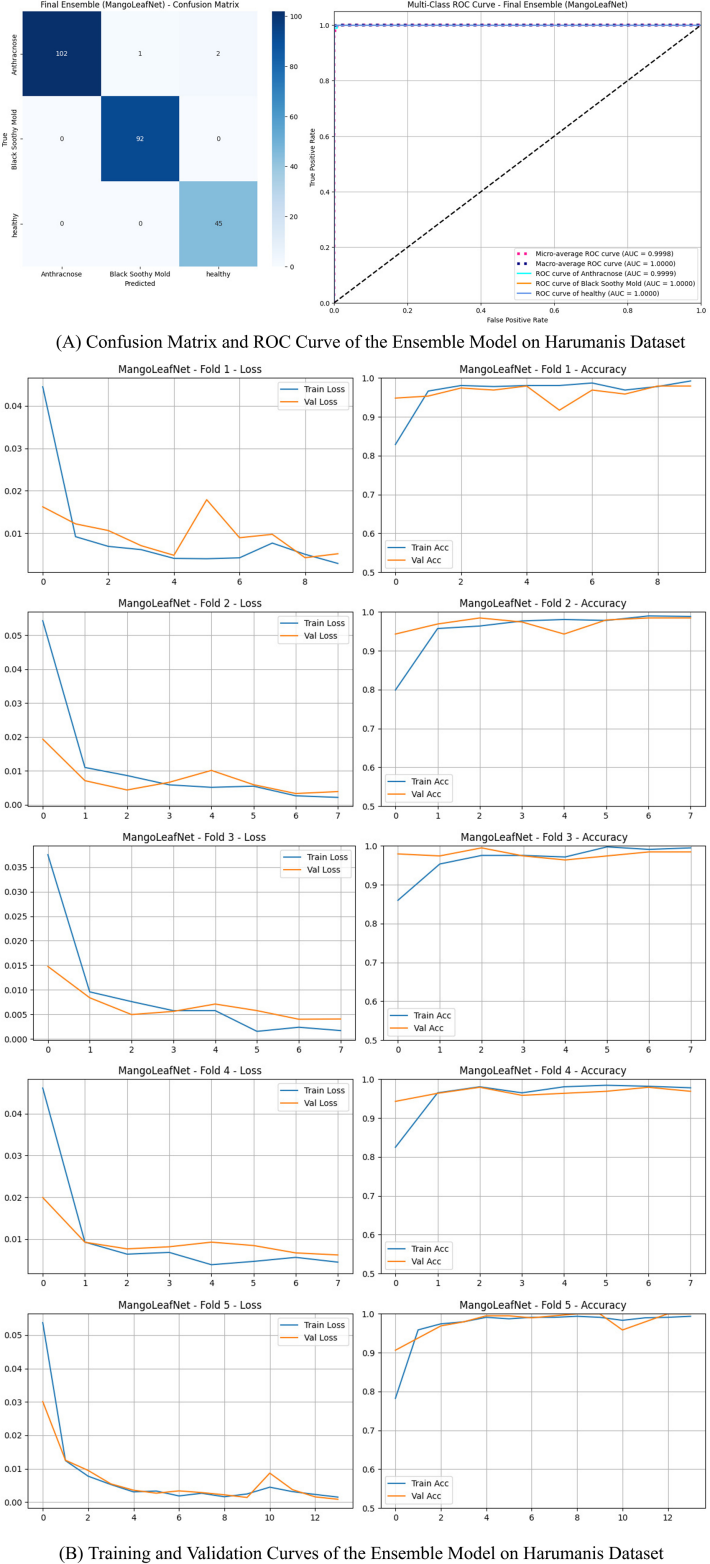
Comprehensive performance visualization – ensemble model (harumanis dataset). **(A)** Confusion Matrix and ROC Curve - Ensemble Model (Harumanis Dataset). **(B)** Training and Validation Curves - Ensemble Model (Harumanis Dataset).

The results include three classes: Anthracnose, Black Sooty Mold, and Healthy. The performance of the ensemble model on the Harumanis Dataset is further depicted in **Figure 5**, showing the confusion matrix and ROC curve.

The model achieved 98.76% accuracy, accurately classifying all three disease classes. Using the DenseNet-121 backbone with sequential Efficient Channel Attention modules, MangoLeafNet-XAI exceeded 98% accuracy, with ECA enhancing discrimination between visually similar illnesses such as Gall Midge and Die Back. Focal Loss addressed class imbalance, yielding stable accuracy and recall. The model was stable in its performance across three geographically disparate datasets, employing pathologically relevant features for predictions validated through Grad-CAM and LIME. Table VII shows MangoLeafNet-XAI as a new state of the art in accuracy, interpretability, and cross-dataset stability.

### 4.3 Discussion

The experimental results provide valuable information regarding the performance of pre-trained architectures in classifying mango leaf disease. The superior performance of MangoLeafNet-XAI is attributed to the synergistic effect of its core components. The DenseNet-121 backbone provides rich, multi-scale feature representations through dense connectivity. However, the main performance improvement comes from the ECA modules, which implement a channel attention mechanism without reducing dimensionality. This preserves cross-channel information that is often lost in traditional attention blocks, allowing the model to distinguish subtle morphological similarities in diseases such as Gall Midge and Die Back. Additionally, the use of Focal Loss shifts the optimization focus from easily classified healthy tissue to complex, overlapping disease boundaries. This combination enables the model to outperform deeper networks such as VGG-16, which lack attention mechanisms, and lightweight networks such as MobileNetV2, which lack sufficient depth for complex feature extraction. Accuracy–Complexity Trade-off More complex models such as VGG16, DenseNet121, and ResNet50, on data sets such as MLDID and Harumanis, achieved higher accuracies since they have the ability to learn hierarchical features. The technical definition of classification accuracy is:

(17) $$\text{Accuracy}=\frac{TP+TN}{TP+TN+FP+FN}$$

In Equation 17, TP, TN, FP, and FN stand for true positives, true negatives, false positives, and false negatives, respectively. Although they perform well, such models are not fit for edge deployment due to the high computational requirements.

Unstable Results of Light-Weight Models All light-weight designs like SqueezeNet, ShuffleNet, and MobileNet variants all produced unstable results. ShuffleNet V2 performed well on the Mango Leaf Disease dataset but broke down severely on MLDID and Harumanis. SqueezeNet too performed well on some datasets but failed on others, indicating vulnerability to variation in background, symptom morphology, and leaf imaging.

Dataset Dependency and Generalization Performance strongly depends on the dataset. VGG16 performed poorly on the Mango Leaf Disease dataset while excelling on others. F1-score is defined as Equations 18, 19:

(18) $$\text{F}1\text{-score}=2\times \frac{\text{Precision}\times \text{Recall}}{\text{Precision}+\text{Recall}}$$

Where,

(19) $$\text{Precision}=\frac{TP}{TP+FP},\text{ Recall}=\frac{TP}{TP+FN}.$$

High F1-scores of deep models indicate strong discriminative power, whereas lightweight models show instability.

Practical Deployment Deep models are suitable for centralized, resource-rich systems, while EfficientNetB0 balances accuracy and efficiency for mobile or IoT monitoring. Future research should focus on designing robust lightweight models, integrating explainable AI, and employing domain adaptation and cross-dataset training to enhance generalization.

## 5 Model explainability

To make clinical decision-making transparent, the integrated explainable AI (XAI) methods into MangoLeafNet-XAI. Since deep convolutional neural networks are inherently black-box, interpretability is essential for diagnosing agricultural diseases, where predictions must be accompanied by useful information. Local Interpretable Model-Agnostic Explanations (LIME) and Gradient-weighted Class Activation Mapping were two complimentary explanation techniques used.

Grad-CAM backpropagates gradients from the output layer to intermediate convolutional feature maps, resulting in class-discriminative localization maps. The important weight of feature map ***k*** for an input picture ***x*** and target class ***c*** is determined by global average pooling of the gradients, as shown in Equation 20:

(20) $$\alpha_{k}^{c}=\frac{1}{Z}\sum_{i}\sum_{j}\frac{\partial y^{c}}{\partial A_{ij}^{k}}$$

In Equation 21, $A^{k}\in {\mathbb{R}}^{H\times W}$ is the feature map at channel k, Z is the normalization factor (spatial dimensions), and $y^{c}$ is the score for class c. Next, the class activation map is shown as follows:

(21) $$L_{\text{Grad-CAM}}^{c}=\text{ReLU}\;\left(\sum_{k}\alpha_{k}^{c}A^{k}\right)$$

MangoLeafNet-XAI ‘s attention to pathogen-induced lesions rather than irrelevant background may be visually verified thanks to this heatmap, which displays the discriminative areas in infected mango leaves.

To achieve instance-level interpretability, we concurrently implemented LIME. With d being the number of segments, LIME transforms the input picture x into a collection of interpretable superpixels $z\in {\left\{0,1\right\}}^{d}$. Next, the following goal is minimized in order to optimize a locally faithful surrogate model g(z):

(22) $$ℒ\left(f,g,\pi_{x}\right)+\text{Ω}\left(g\right)$$

In Equation 22, f represents the original MangoLeafNet-XAI model, $\pi_{x}$ is a proximity measure that takes into account locality around instance x, and Ω(g) penalizes model complexity in order to ensure interpretability. By doing this, LIME identifies locations that impacted MangoLeafNet-XAI ‘s prediction- those that are contradicting (red) and supporting (green).

By combining Grad-CAM’s global feature attribution with LIME’s local surrogate explanations, our framework provides both dataset-level and instance-level transparency. Importantly, the qualitative overlays confirmed that MangoLeafNet-XAI consistently focused on symptomatic leaf regions such as necrotic spots, canker lesions, and midrib damage.

### 5.1 Model attention via Grad-CAM

Gradient-weighted Class Activation Mapping, a popular visualization method for locating class-discriminative areas in convolutional feature maps, was used to shed light on MangoLeafNet-XAI’s decision-making process (Selvaraju et al., 2017). Grad-CAM uses the gradients entering the final convolutional layer to emphasize semantically significant areas that are most important for class prediction, in contrast to pixel-level saliency maps, which might result in noisy attributions.

Let $y^{c}$ represent the pre-softmax score for class c and $A^{k}\in {\mathbb{R}}^{H\times W}$ represent the k-th feature map of the final convolutional layer for a given input image x and target class c. The global average of the gradients is used to calculate the important weight $\alpha_{k}^{c}$ for feature map k, as shown in Equation 13:

(23) $$\alpha_{k}^{c}=\frac{1}{Z}\sum_{i}\sum_{j}\frac{\partial y^{c}}{\partial A_{ij}^{k}}$$

The spatial normalization factor is represented by Z=H×W. Next, we define the Grad-CAM heatmap as follows:

(24) $$L_{\text{Grad-CAM}}^{c}=\text{ReLU}\;\left(\sum_{k}\alpha_{k}^{c}A^{k}\right)$$

In Equation 24, the ReLU function produces localized visual explanations by ensuring that only traits that positively impact the target class are kept.

**Figure 6:**
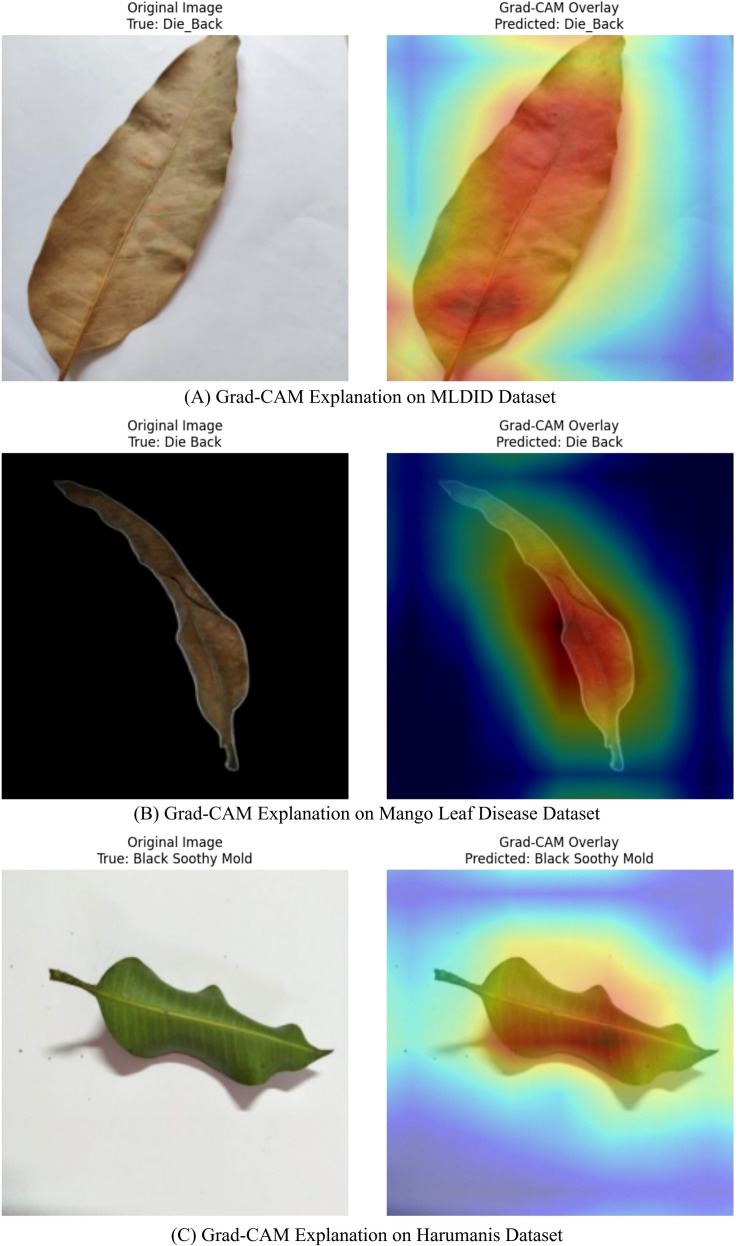
Comprehensive grad-CAM visualization – ensemble model (MLDID, mango leaf disease, and harumanis datasets). **(A)** Grad-CAM Explanation on MLDID Dataset. **(B)** Grad-CAM Explanation on Mango Leaf Disease Dataset. **(C)** Grad-CAM Explanation on Harumanis Dataset.

In pathogenic mango leaves, Grad-CAM was able to effectively suppress background noise and prioritize important lesion regions such as necrotic patches, midrib infections, and cankered tissue, confirming that MangoLeafNet-XAI relied on actual pathological evidence. **Figure 6** shows correct anthracnose detection, where red-yellow heatmap regions point to necrotic edges, with blue regions having negligible effect. Bacterial canker highlighting midrib lesions and chlorotic patches identified by experts. Bacterial spot is identified by characteristic brown lesion activity, in complete agreement with agricultural diagnostic characteristics and affirming correct classification.

In addition, the model’s focus and symptomatic regions determined by experts were in great agreement, according to qualitative overlays of Grad-CAM heatmaps. For example, Grad-CAM highlighted irregular necrotic boundaries and chlorotic lesions, respectively, which are characteristic diagnostic features for bacterial canker and anthracnose. Such targeted focus strengthens the model’s reliability in practical agricultural diagnostics by highlighting its capacity to rank clinically significant variables while eliminating noise.

### 5.2 Local model-agnostic explanations

Despite highlighting global class-discriminative zones, Grad-CAM still relies on feature maps and internal gradients (Ribeiro et al., 2016). In addition, we used a perturbation-based method called Local Interpretable Model-Agnostic Explanations (LIME), which creates locally accurate surrogate models based on individual predictions. Because LIME offers fine-grained interpretability independent of the MangoLeafNet-XAI underlying architecture, it is very useful in agricultural disease diagnoses.

In formal terms, LIME optimizes an interpretable surrogate model g∈G (usually sparse linear) given the original model f, an input picture x, and a perturbed neighborhood distribution $\pi_{x}$ by solving:

(25) $$\xi \left(g\right)=\text{arg}\underset{g\in G}{\text{min}}\;ℒ\left(f,g,\pi_{x}\right)+\text{Ω}\left(g\right)$$

In Equation 25, Ω(g) penalizes model complexity to ensure interpretability, and $ℒ\left(f,g,\pi_{x}\right)$ quantifies the fidelity of surrogate g to the complicated model f within the immediate region of x. By selectively masking or modifying the original image’s superpixels, perturbations $x^{\prime }$ are created, which enable LIME to calculate the contribution of each interpretable area to the final forecast.

**Figure 7:**
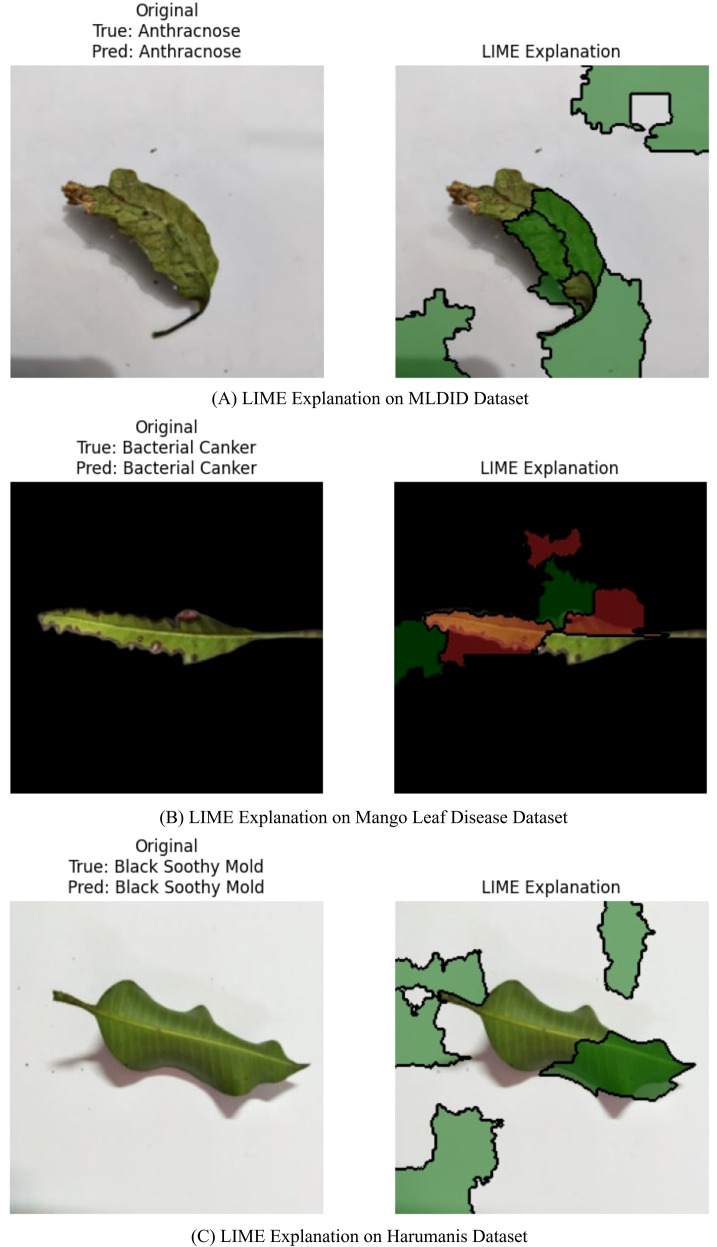
Comprehensive LIME visualization – ensemble model (MLDID, mango leaf disease, and harumanis datasets). **(A)** LIME Explanation on MLDID Dataset. **(B)** LIME Explanation on Mango Leaf Disease Dataset. **(C)** LIME Explanation on Harumanis Dataset.

**Figure 7** illustrates the MangoLeafNet-XAI model’s interpretation of an anthracnose-affected leaf using LIME. The model precisely predicts the disease, with green superpixels extracting necrotic regions validating its prediction and red for contradicting features. Detection of bacterial canker is demonstrated, where symptomatic lesions are represented by green superpixels and healthy areas by red ones, validating diagnostic accuracy. A healthy leaf displays low disease-related activation, where green patches validate the precision of the model’s predictions and provide crucial visual evidence that addresses the black-box concern in agricultural AI. By demonstrating that the network disregards non-predictive artifacts such as shadows or soil and concentrates solely on meaningful pathological cues like midrib damage or chlorotic regions, we show that the model’s high accuracy arises from authentic disease characteristics rather than dataset-specific biases and absence of infection markers.

The comparative analysis in **Table 10** confirms the tremendous progress achieved by the MangoLeafNet-XAI model over earlier state-of-the-art methods for mango leaf disease classification. Earlier approaches such as AlexNet-based model by Rao et al. (2021) only attained a 89.00% accuracy and fell short in poor illumination and occluded leaf imaging conditions. Although LeafNet of Rizvee et al. (2023) was as accurate as 98.55%, it was not explainable and was only validated on homogeneous datasets. Similarly, deep models such as InceptionV3 by Varma et al. (2025) were not suited for resource-scarce environments. In contrast, MangoLeafNet-XAI combines adaptive channel attention with the parameter-efficient structure of DenseNet and adds Grad-CAM and LIME within an Explainable AI framework and yields transparent, coherent, and exceedingly generalizable disease diagnosis across different datasets.

**Table 10:** Performance comparison of MangoLeafNet-XAI with existing methods.

Ref.	Model	Dataset	Accuracy	Precision	Recall	F1-score	XAI
Rizvee et al. (2023)	LeafNet	MangoleafBD	98.55	98.5	98.45	98.47	✗
Prabu and Chelliah (2022)	CNN + CROLFD	Mango Leaf (India)	–	–	98.42	–	✗
Varma et al. (2025)	InceptionV3 (Transfer Learning)	PlantVillage	99.87	–	–	–	✗
Rao et al. (2021)	AlexNet (Transfer Learning)	Custom Mango	89.00	–	–	–	✗
Mahbub et al. (2023)	LCNN	–	98	97.6	97.5	97.5	✗
Singh et al. (2019)	MCNN	–	97.13	–	–	–	✗
Vijay and Pushpalatha (2023)	EfficientNetB4 + Custom CNN	–	93.01	93.23	94	93.07	✗
Proposed	**MangoLeafNet-XAI (Ensemble)**	**MLDID**	**98.83**	**98.87**	**98.83**	**98.83**	**✓ (Grad-CAM, LIME)**
	**MangoLeafNet-XAI (Ensemble)**	**Mango Leaf Disease**	**98.09**	**98.21**	**98.32**	**98.25**	**✓ (Grad-CAM, LIME)**
	**MangoLeafNet-XAI (Ensemble)**	**Harumanis**	**98.76**	**98.22**	**99.05**	**98.61**	**✓ (Grad-CAM, LIME)**

The values in bold represent the performance results of our proposed model, MangoLeafNet-XAI (Ensemble), across different datasets.

## 6 Limitations and future work

Though its high accuracy has been attained, the model remains light-sensitive, affected by occlusions and background noise, which can limit its application in field, unstructured environments. High-quality, pure training datasets also potentially reduce its ability for early detection of disease signs under noisy field conditions. Though existing tools for explainability offer some insights, expert validation is required to ensure practical usability, and computational demands can prove challenging for low-power edge devices. Future research needs to target improving generalization through training over many domains, the integration of multimodal data like spectral imaging and environmental input, and the design of effective dynamic neural architectures. Developing a large, standardized public corpus of mango leaf disease data would incentivize collaboration and support reproducible innovation in agricultural AI.

## 7 Conclusion

MangoLeafNet-XAI is a new deep learning framework for solving key issues in automatic mango leaf disease identification. MangoLeafNet-XAI integrates Efficient Channel Attention modules with DenseNet-121 as the backbone and has achieved state-of-the-art accuracy of 98.00 on three public datasets with only 6.9 million parameters for computational efficiency. Through extensive 5-fold cross-validation and ensemble averaging, the model demonstrates good generalizability and reliability. Techniques of Explainable AI such as Grad-CAM and LIME offer transparency by ensuring that predictions are made based on real pathological features and not on spurious correlations. This interpretability fosters trust and adoption in the real world for precision agriculture, establishing a new standard for trustworthy, scalable, and transparent plant disease diagnosis that supports sustainable and efficient crop management.

## Data Availability

The original contributions presented in the study are included in the article/supplementary material. Further inquiries can be directed to the corresponding author.
